# Supplementary material for: Transcriptomic Insights into Mechanisms of Early Seed Maturation in the Garden Pea (Pisum sativum L.)
Source: Cells. 2020 Mar 23;9(3):779. doi: 10.3390/cells9030779 (PMC7140803; doi:10.3390/cells9030779)

Independent Component Analysis on module data (dmap)

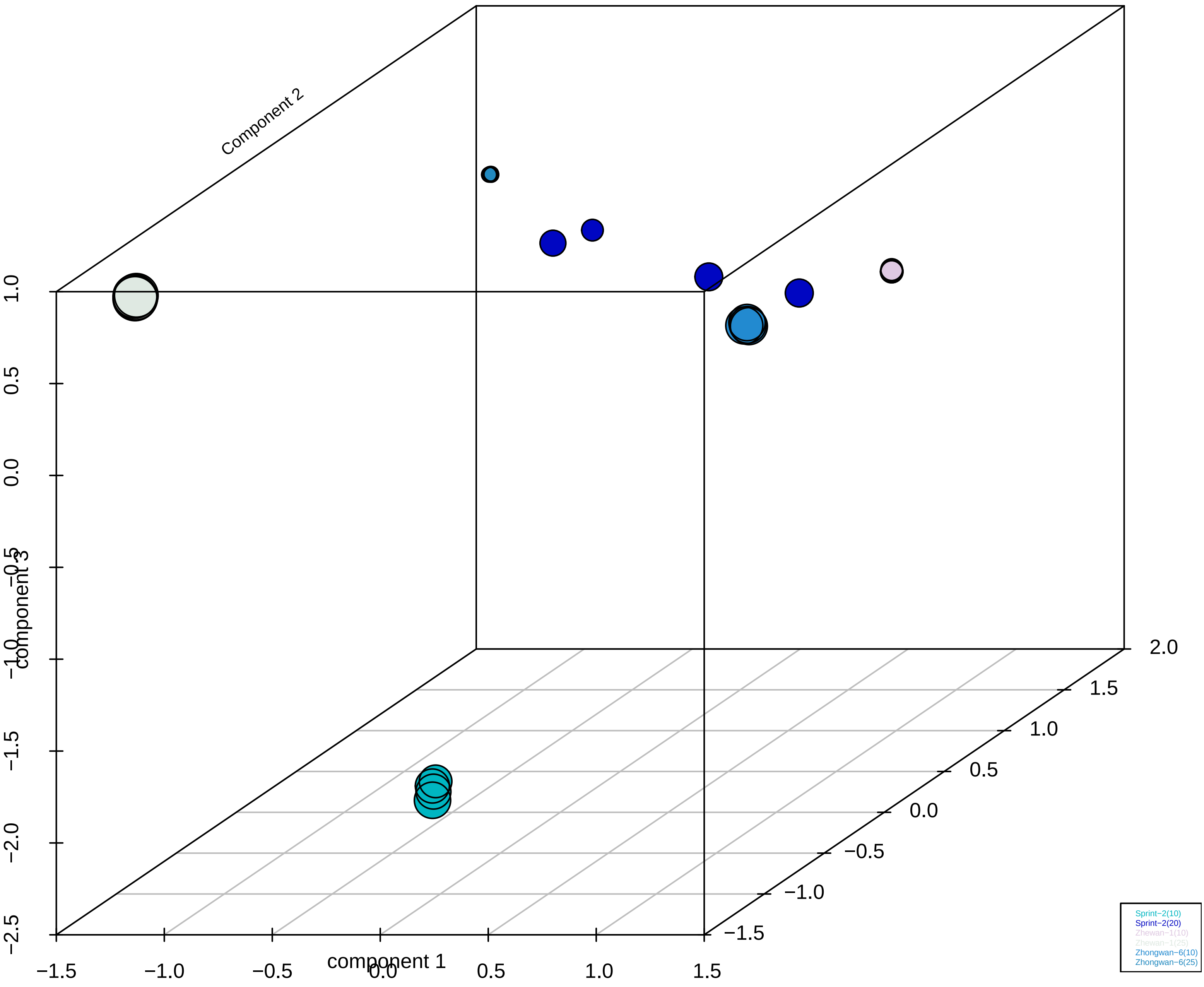

Independent Component Analysis on module data (dmap)

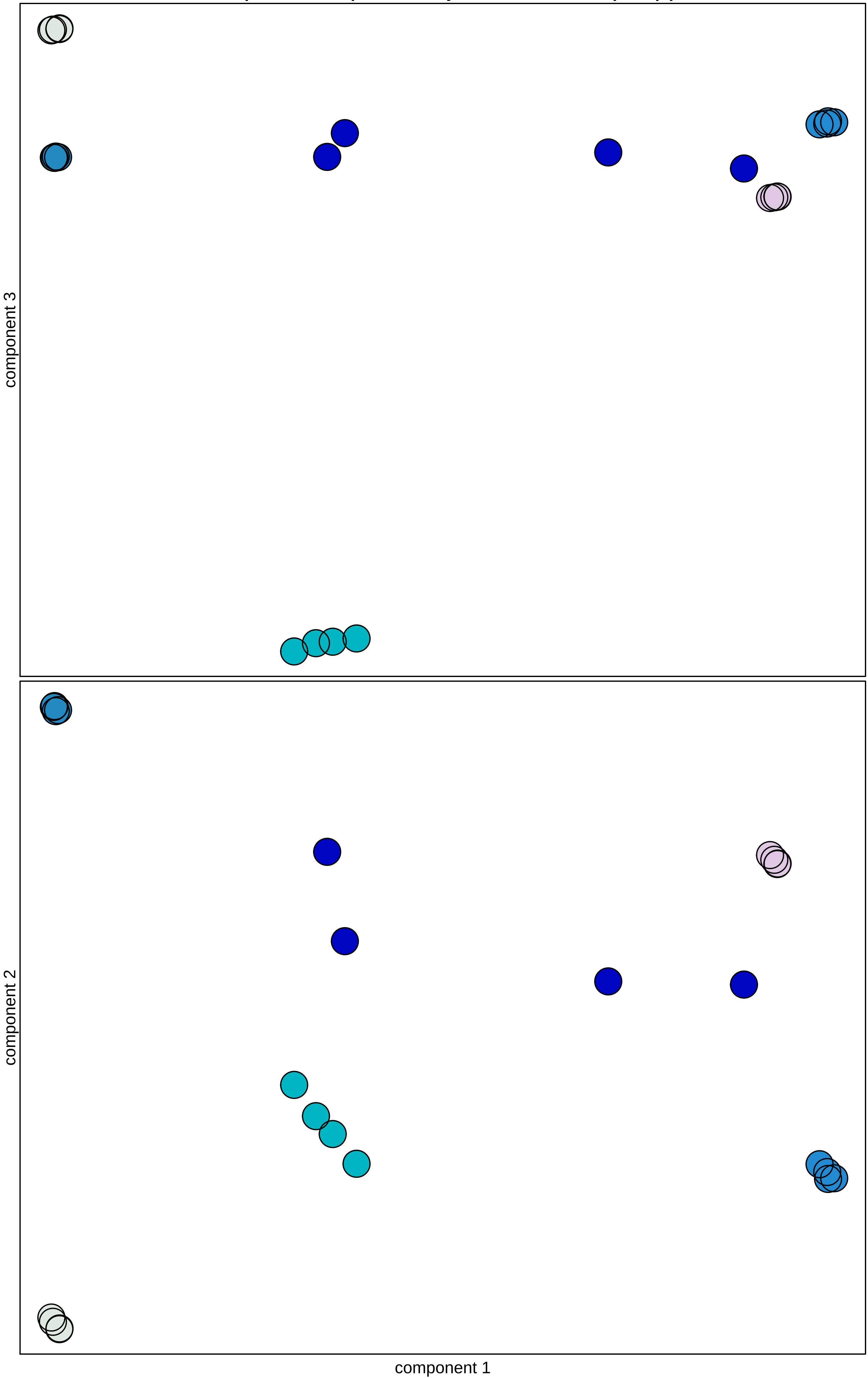

Independent Component Analysis on module data (dmap)

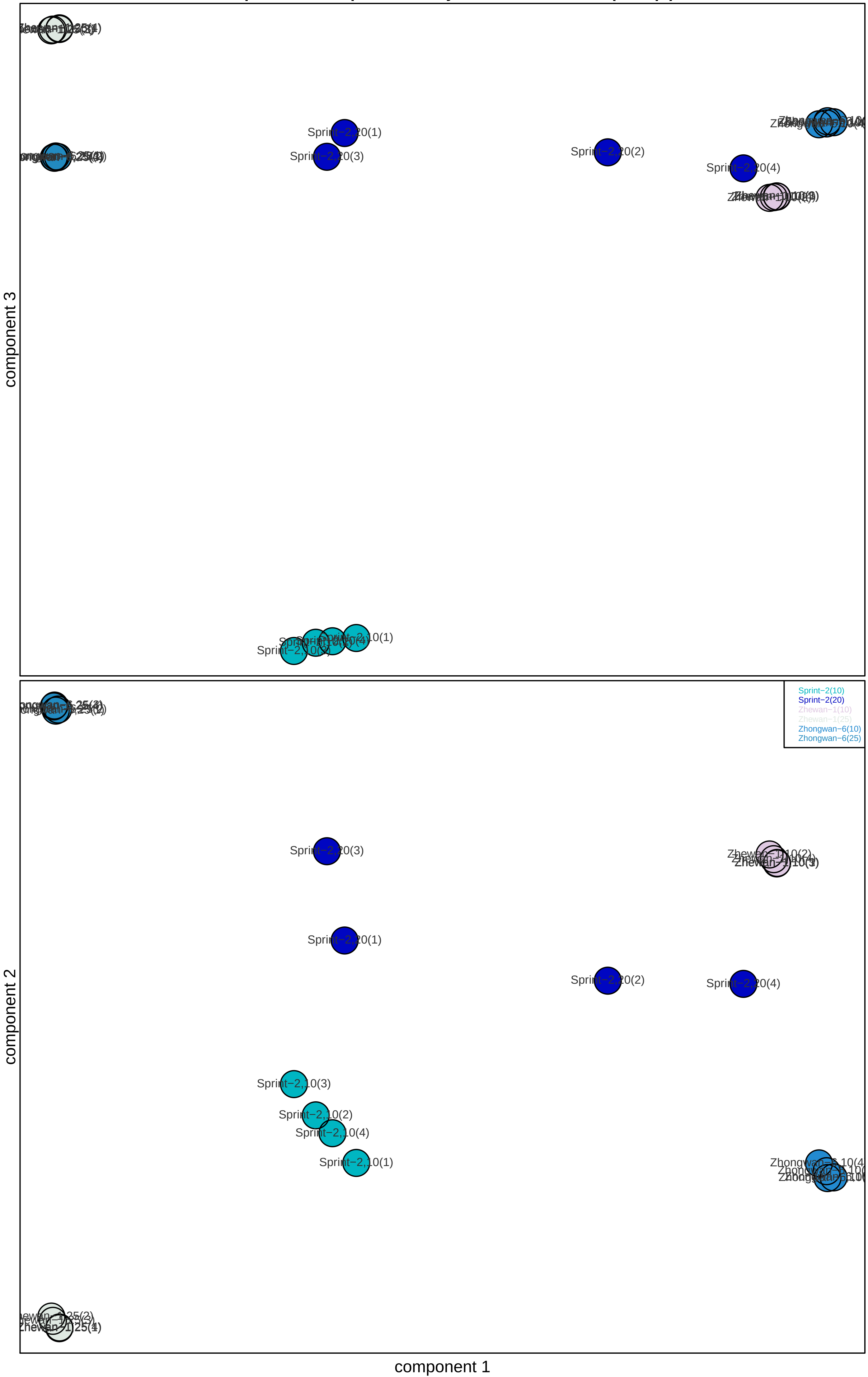

Independent Component Analysis on metagene data

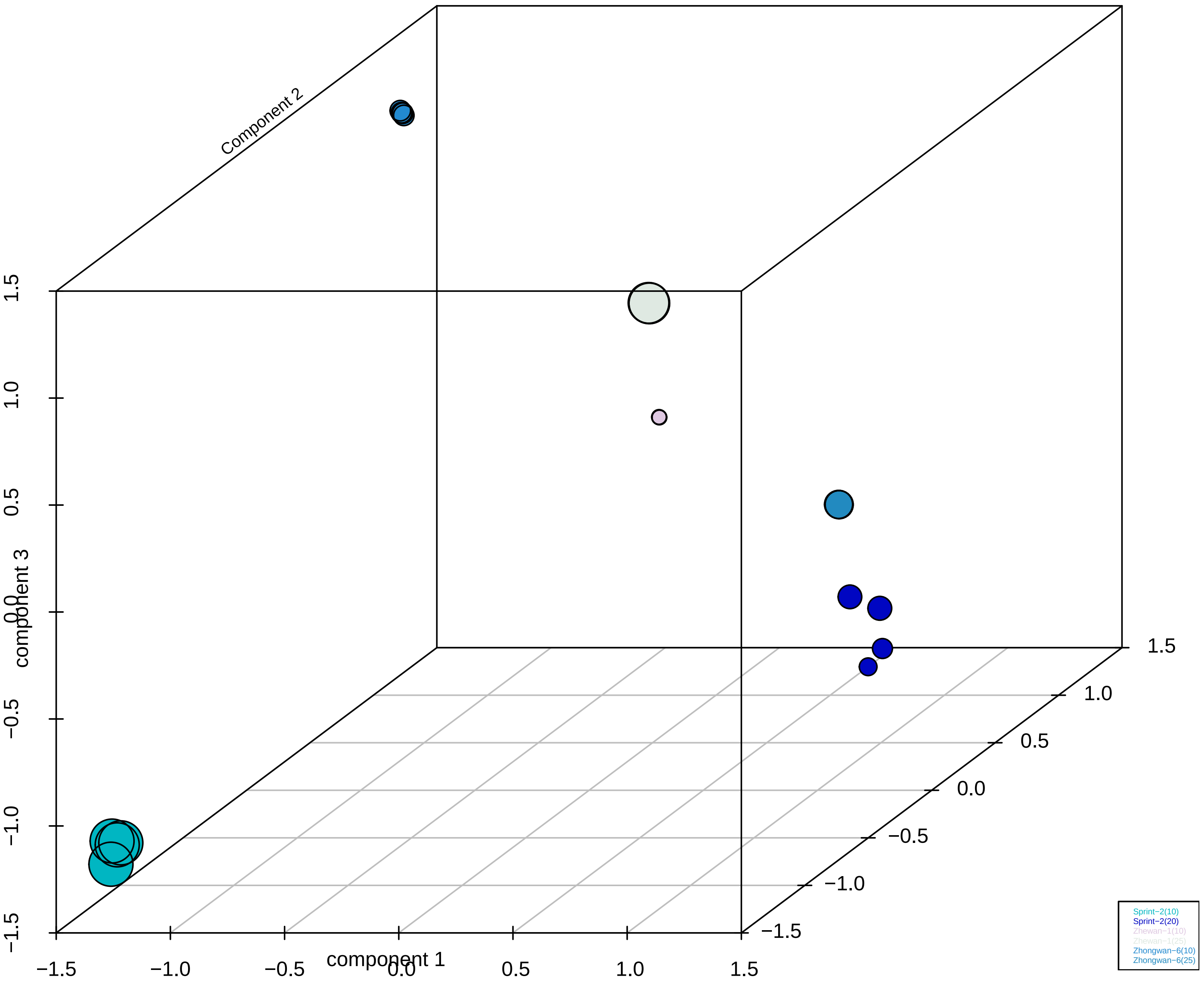

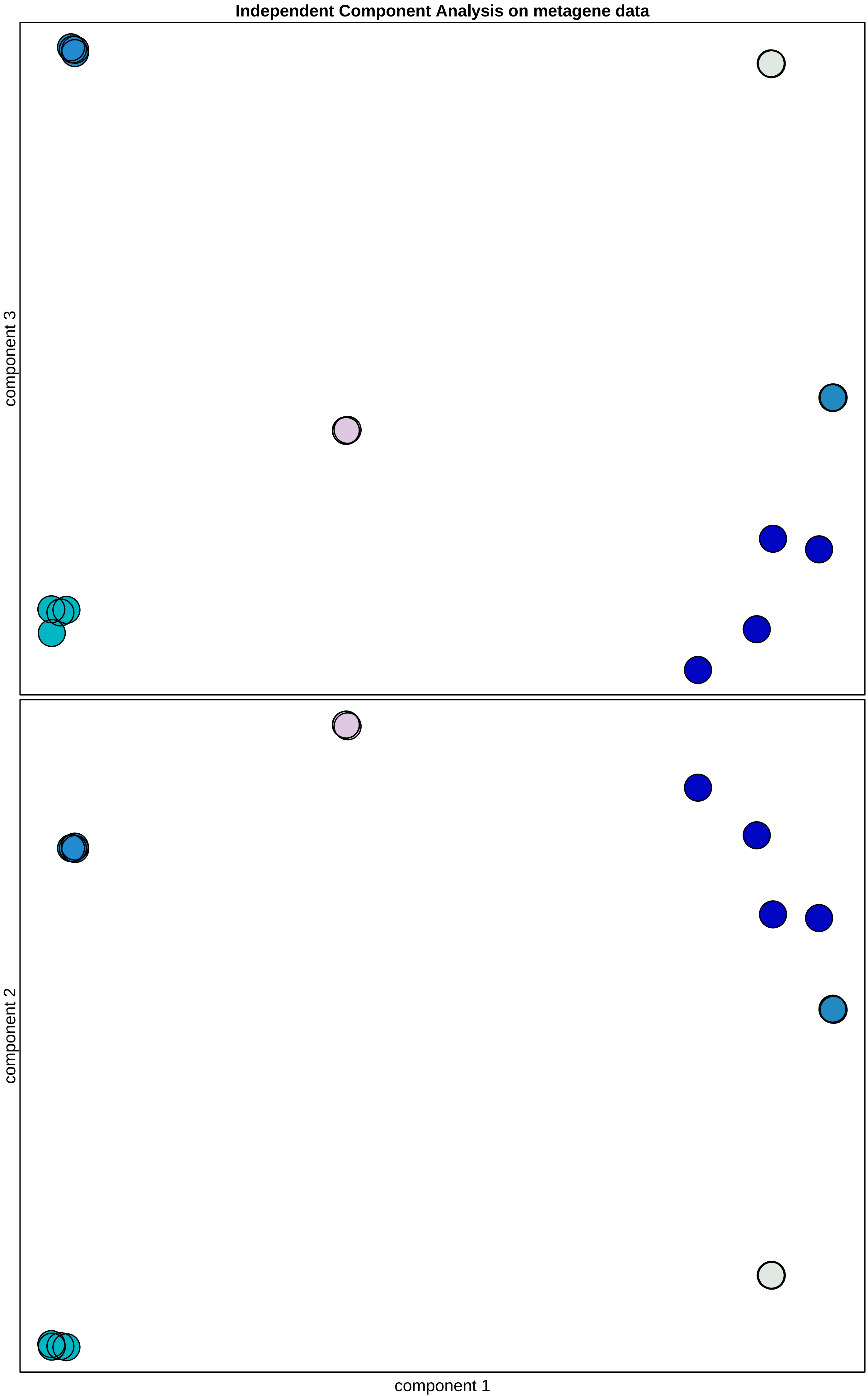
Independent Component Analysis on metagene data
component 3
component 2
component 1

Independent Component Analysis on metagene data

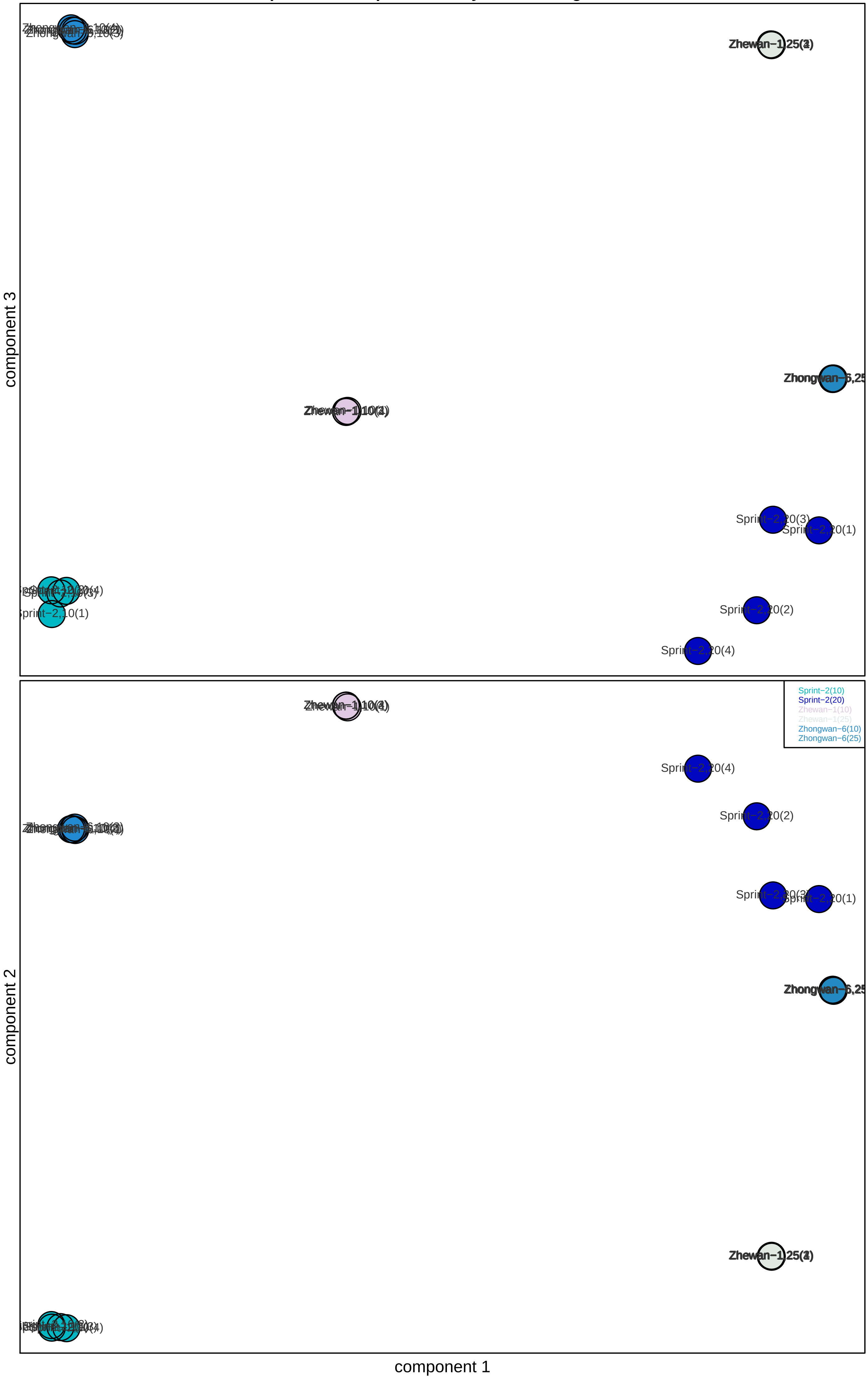

Supplement: Supplementary file 1 [file cells-09-00779-s001.zip › Supplementary materials/FileS2.zip!/FileS1/Sample Similarity Analysis/Component Analysis.pdf]
